# Bulbar Conjunctival Thickness in Eyes with Pseudoexfoliation Measured by Anterior Segment OCT

**DOI:** 10.18502/jovr.v20.15341

**Published:** 2025-05-05

**Authors:** Mahla Shadravan, Shahin Yazdani, Bahareh Kheiri, Farideh Sharifipour, Azadeh Doozandeh, Mohammad Mehdi Hatami, Neda Einollahi

**Affiliations:** ^1^Clinical Research Development Unit, Shafa Hospital, Kerman University of Medical Sciences, Kerman, Iran; ^2^Ocular Tissue Engineering Research Center, Research Institute for Ophthalmology and Vision Science, Shahid Beheshti University of Medical Sciences, Tehran, Iran; ^3^Ophthalmic Research Center, Research Institute for Ophthalmology and Vision Science, Shahid Beheshti University of Medical Sciences, Tehran, Iran; ^4^Department of Ophthalmology, Labbafinejad Medical Center, Shahid Beheshti University of Medical Sciences, Tehran, Iran

**Keywords:** Anterior Segment Optical Coherence Tomography, Conjunctival Thickness, Pseudoexfoliation

## Abstract

**Purpose:**

Eyes with pseudoexfoliation demonstrate fragile intraocular structures. The current study evaluated conjunctival thickness in pseudoexfoliation eyes as compared to a control group.

**Methods:**

In this cross-sectional study, patients with pseudoexfoliation and an age-matched control group underwent measurement of bulbar conjunctival thickness in the superior and temporal quadrants, 1, 2, and 3 mm posterior to the limbus using anterior segment OCT.

**Results:**

A total of 140 eyes of 140 subjects including 68 eyes of 68 patients with pseudoexfoliation and 72 eyes of 72 control subjects without pseudoexfoliation were studied. Both study groups were divided into two subgroups – those using glaucoma drops versus medication-free eyes. Total conjunctival thickness in pseudoexfoliation eyes was significantly lower than control eyes across all comparisons. Conjunctival thickness in the superior quadrant 1, 2, and 3 mm from the limbus was 177.62 ± 41.30, 235 ± 48.41, and 231.40 ± 49.81 µm, respectively in the pseudoexfoliation group versus 207.49 ± 48.92, 265.67 ± 52.66, and 262.74 ± 59.43 µm in the control group (*P* < 0.001, < 0.001, and 0.001, respectively). In the temporal quadrant, conjunctival thickness 1, 2, and 3 mm from the limbus was 193.62 ± 46.97, 198.19 ± 54.67, and 178.59 ± 57.90 µm, respectively in the pseudoexfoliation group versus 213.76 ± 47.06, 224.50 ± 56.24, and 210.26 ± 63.70 µm in the control group (*P* = 0.012, 0.006, and 0.003, respectively).

**Conclusion:**

Conjunctival thickness was significantly less in pseudoexfoliation eyes than an age-matched control group which supports clinical observations of thinner and more fragile conjunctiva in these eyes with implications for glaucoma surgery.

##  INTRODUCTION

Pseudoexfoliation (PXF) syndrome is considered a systemic, age-related disease with typically bilateral, but often asymmetrical, ocular manifestations.^[1,2]^ PXF is a significant risk factor for glaucoma and the most common cause of secondary open-angle glaucoma.^[3]^ Ocular manifestations of PXF predominantly involve the anterior segment, the hallmark of which is deposition of grayish-white fibrogranular material but may also include pigment dispersion, inadequate mydriasis, zonular fragility and disruption, phacodonesis, lens subluxation, corneal endothelial decompensation, incompetent blood-aqueous barrier, anterior chamber hypoxia, and posterior synechiae.^[2]^ Studies have also indicated that eyes with PXF tend to have thinner corneas and longer axial lengths.^[4]^

Bulbar conjunctiva and Tenon's layer thickness varies among individuals, influencing the outcomes of conjunctiva-based glaucoma procedures, particularly bleb morphology and function after bleb-forming glaucoma procedures.^[5]^ Conjunctival structure is also of significance regarding bleb-related infections and endophthalmitis. Risk factors for these complications include leakage, which is particularly prevalent in thin-walled blebs, and following the use of anti-fibrotic agents during filtering surgery.^[6]^

In our experience, the bulbar conjunctiva in PXF patients appears to be thin, delicate, and fragile during surgery as compared to non-PXF eyes leading to a higher risk of conjunctiva-related complications during glaucoma surgery. We also speculate that these factors could influence the outcomes of conjunctiva-based glaucoma procedures including but not limited to trabeculectomy.

Anterior segment OCT (optical coherence tomography) may be employed for imaging of the tear film, cornea, conjunctiva, and anterior chamber angle.^[7]^ High-resolution anterior segment OCT can capture detailed cross-sectional images of conjunctival layers, enabling thickness measurements.^[8]^

In this study, we utilized anterior segment OCT to assess conjunctival thickness in eyes with PXF in comparison to an age-matched control group.

##  METHODS

This cross-sectional study was conducted at Labbafinejad Medical Center (LMC), Tehran, Iran. Subjects were recruited from October 2021 to April 2022. The case group comprised of eyes with PXF; an age-matched control group was selected among subjects with no evidence of PXF. As our study was the first to examine conjunctival thickness in patients with PXF, the sample size was determined based on an initial pilot study. With an effect size of 0.65, a significance level of 0.05, a sample size of 68 in either study group would provide a study power of 95%. Only one eye per subject was included in the study. This study adhered to the tenets of the Declaration of Helsinki and was approved by the Research Institute for Ophthalmology and Vision Science at Shahid Beheshti University of Medical Sciences.

Inclusion criteria for enrolling PXF cases were age over 55 years and detection of PXF material in the anterior segment, regardless of the presence of ocular hypertension or glaucomatous optic neuropathy. PXF material was diagnosed on slit-lamp examination by the presence of gray-white fibrogranular deposits on the pupil margin, corneal endothelium, anterior chamber angle, or anterior lens capsule after pupil dilation. Only eyes with virgin conjunctiva were enrolled and other ocular pathologies such as pterygium, conjunctivochalasis, severe dry eye, and high refractive errors were excluded; eyes that had undergone glaucoma surgery or other procedures affecting the conjunctiva were also excluded. The control group included individuals over 55 with and without glaucoma (except PXF glaucoma) with virgin conjunctiva in the absence of other ocular pathologies. Prior clear cornea phacoemulsification cataract surgery was not considered as an exclusion criterion for either study group. Our case and control patients did not use any topical medications except for glaucoma medications. To assess the possible effect of concomitant use of topical glaucoma medications, the study groups were evaluated in two subgroups: those using glaucoma eye drops and those not receiving such medications.

A comprehensive eye examination was conducted for all study subjects including measurement of uncorrected and best corrected visual acuity, anterior segment examination, intraocular pressure measurement using Goldmann applanation tonometry, and fundus examination. Baseline data, including age, sex, and medical, surgical, and family history were recorded.

**Figure 1:**
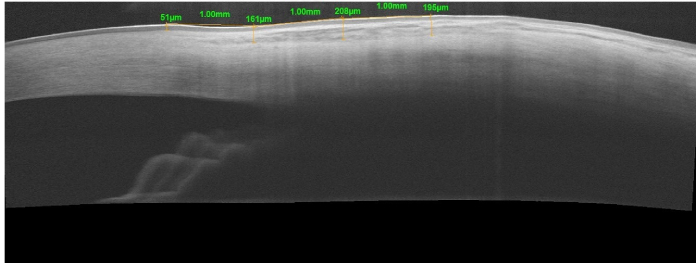
Representative image depicting tissue boundaries and conjunctival thickness measurements.

All subjects underwent measurement of total bulbar conjunctival plus Tenon thickness in the superior and temporal quadrants using a spectral domain OCT machine (RTVue XR 100 Avanti Edition OCT; Optovue Inc. Fremont, CA, USA) employing the anterior segment line protocol of the device. High-quality images were captured and the instrument's software was employed to measure total thickness 1, 2, and 3mm from the external limbus. The thickness of the conjunctiva–Tenon complex was measured from the conjunctival epithelium to the posterior surface of the conjunctival stroma and Tenon's layer. The conjunctival epithelium appears relatively hypo-reflective just below the tear film. Beneath it, the conjunctival stroma is hyper-reflective and Tenon's layer also exhibits hyper-reflectivity similar to the stroma. However, it gradually becomes less reflective just above the distinctly curved reflection of the sclera [Figure 1]. Measurements were obtained by two independent examiners who were unaware of case and control assignments. Data from both examiners showed high agreement (kappa 0.78) and average measurements from both examinations were used for the study.

SPSS 25.00 software was utilized for statistical analysis. Significance was set at 0.05. Descriptive statistics such as mean, standard deviation, frequency, and percentage were used to present data. ANOVA statistical tests were employed to compare mean values after verifying data normality through Q-Q diagrams. Pairwise comparisons of the study groups were conducted using the Bonferroni method. The distribution of gender between groups was compared using Fisher's exact test.

##  RESULTS

A total of 140 eyes of 140 subjects were enrolled in the study. The case group consisted of 68 eyes of 68 patients with PXF including 32 eyes receiving topical glaucoma medications and 36 eyes using no medications. The control group comprised 72 eyes of 72 subjects without PXF, including 47 eyes receiving topical glaucoma medications and 25 eyes using no medications [Table 1]. The mean age of case and control subjects was 69.53 ± 8.45 versus 68.01 ± 7.27 years, respectively (*P* = 0.658).

Across all comparisons, the PXF group demonstrated significantly lower total conjunctival thickness compared to the control group. In the superior quadrant, at distances of 1, 2, and 3 mm from the limbus, the mean total conjunctival thickness was 177.62 ± 41.30, 235 ± 48.41, and 231.40 ± 49.81 µm, respectively in the PXF group which was significantly lower than the corresponding values of 207.49 ± 48.92, 265.67 ± 52.66, and 262.74 ± 59.43 µm in the control group (*P*-values < 0.001, < 0.001, and 0.001, respectively). Similarly, in the temporal quadrant, the mean total conjunctival thickness 1, 2, and 3 mm from the limbus was 193.62 ± 46.97, 198.19 ± 54.67, and 178.59 ± 57.90 µm, respectively in the PXF group which was significantly lower than the corresponding values of 213.76 ± 47.06, 224.50 ± 56.24, and 210.26 ± 63.70 µm in control eyes (*P*-values 0.012, 0.006, and 0.003, respectively) [Table 2]. No significant correlation was observed between age and conjunctival thickness (all *P*-values > 0.05) [Table 3].

To compensate for the possible effect of concomitant use of topical glaucoma medications, the analysis was repeated between study subgroups (i.e., eyes receiving topical glaucoma medications and eyes not using such medications) [Table 2]. Among eyes receiving topical glaucoma medications, PXF cases had significantly lower total conjunctival thickness in the superior quadrant 1, 2, and 3 mm from the limbus. The mean total conjunctival thickness in PXF eyes using glaucoma drops was 171.47 ± 46.11, 217.00 ± 48.05, and 216.66 ± 50.38 µm, respectively which was significantly lower than the corresponding values of 204.51 ± 45.06, 265.94 ± 50.71, and 261.85 ± 50.84 µm in control eyes on topical glaucoma medications (*P*-values: 0.011, < 0.001, 0.002, respectively). PXF eyes not receiving topical glaucoma medications had significantly lower total conjunctival thickness in the temporal quadrant 1, 2, and 3 mm from the limbus. The mean total conjunctival thickness in PXF eyes off topical medications was 189.47 ± 40.69, 197.69 ± 48.89, and 179.94 ± 55.38 µm, respectively which was significantly lower than the corresponding values of 225.60 ± 54.36, 237.00 ± 58.26, and 226.64 ± 63.08 µm in control eyes not using topical medications (*P*-values: 0.022, 0.044, 0.022, respectively).

**Table 1:** Demographic data of the study groups

	**Number**	**Mean age**	**Male**	**Female**	**Axial length**	**Central corneal thickness**
C1	47	68.19	26	21	22.58 ± 0.95	533.28 ± 39.96
C2	25	67.68	12	13	23.37 ± 2.0	528.36 ± 37.6
X1	32	68.83	22	10	23.26 ± 1.41	515.45 ± 44.03
X2	36	70.08	23	13	22.88 ± 1.38	525.16 ± 42.69
C1, control using glaucoma medications; C2, control without glaucoma medications; X1, PXF using glaucoma medications; X2, PXF without glaucoma medications						

**Table 2:** Bulbar conjunctival thickness (µm) in PXF versus control groups

	**Study group**								
	**C1**		**C2**		**X1**		**X2**		
	**Mean**	**Standard deviation**	**Mean**	**Standard deviation**	**Mean**	**Standard deviation**	**Mean**	**Standard deviation**	***P*** -value*
supT.T1mm	204.51	45.06	213.08	56.01	171.47	46.11	183.08	36.28	< 0.001
supT.T2mm	265.94	50.71	265.16	57.22	217.00	48.05	251.06	43.36	< 0.001
supT.T3mm	261.85	50.84	264.40	74.09	216.66	50.38	244.50	46.11	0.001
temT.T1mm	207.47	41.95	225.60	54.36	198.28	53.45	189.47	40.69	0.012
temT.T2mm	217.85	54.59	237.00	58.26	198.75	61.32	197.69	48.89	0.006
temT.T3mm	201.55	62.96	226.64	63.08	177.06	61.46	179.94	55.38	0.003
C1, control eyes using glaucoma medications; C2, control eyes without glaucoma medications; X1, PXF eyes using glaucoma medications; X2, PXF eyes without glaucoma medications									
${\;}^{*}$ All cases versus all controls; ANOVA test (pairwise comparison based on Bonferroni correction)									

**Table 3:** Correlations between age and conjunctival thickness

Total thickness Superior 1 mm	Pearson correlation	–0.106
	*P*-value	0.431
Total thickness Superior 2 mm	Pearson correlation	0.006
	*P*-value	0.964
Total thickness Superior 3 mm	Pearson correlation	–0.030
	*P*-value	0.823
Total thickness Temporal 1 mm	Pearson correlation	–0.102
	*P*-value	0.452
Total thickness Temporal 2 mm	Pearson correlation	–0.233
	*P*-value	0.082
Total thickness Temporal 3 mm	Pearson correlation	–0.249
	*P*-value	0.062

##  DISCUSSION

Anterior segment OCT captures high-resolution cross-sectional images of tissues *in vivo*. Several studies have utilized this modality to assess corneal thickness,^[2]^ tear film,^[7]^ and scleral thickness^[9]^ and to determine the thickness of the bulbar conjunctiva and Tenon layer.^[5,8]^ To the best of our knowledge, our study is the first to investigate conjunctival thickness using anterior segment OCT in eyes with PXF. We found that eyes with PXF exhibited significantly thinner conjunctiva as compared to an age-matched control group. This conclusion supports our observations that PXF patients have thinner and more fragile conjunctiva.

Assessment of anterior segment parameters in PXF eyes can help improve outcomes of glaucoma surgery. More specifically, conjunctival thickness and the tissue response to conjunctiva-based glaucoma surgery for PXF glaucoma is important in preventing intraoperative complications such as conjunctival trauma during dissection and handling, and suture-related issues such as buttonholes and needle track leaks. Taking conjunctival structure and resilience into consideration also helps reduce postoperative complications such as hypotony and choroidal effusions by taking precautions during surgery and modifying surgical techniques.

Zhang et al measured bulbar conjunctival thickness using SD-OCT in healthy Chinese subjects and found that total conjunctival thickness was negatively correlated with age.^[8]^ Ferna´ndez-Vigo et al also investigated the impact of age, sex, and refractive error on conjunctival and tenon's capsule thickness using SS-OCT. They found a negative correlation between age and conjunctival and tenon's capsule thickness.^[10]^ However, in the current study we found no significant correlation between age and conjunctival thickness; this finding may be explained by the fact that the age range in our study was not as wide as in the aforementioned reports. In another study, Zhang et al employed anterior segment OCT to visualize and measure the bulbar conjunctiva.^[11]^ Their study consisted of three groups: enucleated porcine eyes, glaucoma eyes undergoing trabeculectomy, and normal eyes. In normal eyes, the mean total conjunctival thickness was 238.8 µm inferotemporally at a distance of 3–5 mm from the limbus. These values are comparable to those obtained in the current study: in the subgroup of normal eyes not using medications, the mean total conjunctival thickness was 226.64 µm in the temporal quadrant, 3 mm from the limbus.

The impact of topical anti-glaucoma medications on conjunctival thickness has been assessed in patients with primary open-angle glaucoma by Tekin et al, who reported that glaucoma drops may influence conjunctival thickness and potentially diminish surgical success by compromising bleb function and morphology.^[12]^ To overcome the confounding effect of these drops on conjunctival thickness, we divided our case and control groups into two subgroups based on the use of topical glaucoma medications. The major findings of our study were replicated in this analysis and in both subgroups, total conjunctival thickness was significantly lower in eyes with PXF.

As mentioned earlier, none of the previous investigations have measured conjunctival thickness in PXF patients using OCT; however, certain studies have evaluated anterior segment parameters in other conditions using OCT. Francoz et al utilized SD-OCT to measure the thickness of corneal, limbal, and bulbar conjunctival epithelium.^[13]^ They reported altered limbal and bulbar conjunctival epithelium in dry eye patients and those using IOP-lowering eye drops. However, the impact of aging appeared to be negligible. Kommula et al used anterior segment SD-OCT for the measurement of scleral thickness in normal Indian eyes.^[9]^ They measured the sclera and conjunctiva as one complex focusing on scleral thickness; in our study, we measured total conjunctival thickness separately. However, our approach to the demarcation of the anterior boundary of the conjunctiva was similar to that adopted by the mentioned investigators.

Imaging modalities other than OCT have been utilized in PXF eyes. Using Scheimpflug imaging, Gunes et al assessed anterior segment and biometric parameters in PXF eyes.^[4]^ These authors concluded that eyes with PXF exhibited thinner corneas and longer axial length compared to healthy controls and that due to potential complications of cataract surgery and the greater risk of glaucoma in PXF eyes, evaluation of anterior segment parameters is of significant importance.

Although our study employed a matched control group to overcome confounders and had an acceptable sample size, we are aware of certain limitations. Many of our patients were unsure of the exact time of prior phacoemulsification cataract surgery or failed to give an accurate medication history (name or class of medication), preventing us from reporting such details.

In summary, in the current study, total conjunctival thickness in PXF eyes with virgin conjunctiva was found to be lower than in an age-matched control group regardless of the use of topical glaucoma medications. These findings confirm clinical observations of thinner and more fragile conjunctiva in these eyes and have implications for conjunctiva-based glaucoma procedures. Surgeons need to be cautious during manipulation of the conjunctiva in PXF eyes to avoid unwanted trauma leading to excessive openings or tears and buttonholes and also choose to be more conservative in the use of anti-fibrotic agents.

##  Financial Support and Sponsorship

None.

##  Conflicts of Interest

None.
